# Omega-3 Polyunsaturated Fatty Acid Levels in Maternal and Cord Plasma Are Associated with Maternal Socioeconomic Status

**DOI:** 10.3390/nu15204432

**Published:** 2023-10-19

**Authors:** Alexandra Hergenrader, Matthew VanOrmer, Rebecca Slotkowski, Maranda Thompson, Alyssa Freeman, Olivia Paetz, Sarah Sweeney, Lauren Wegner, Khadijjta Ali, Nicole Bender, Ridhi Chaudhary, Melissa Thoene, Corrine Hanson, Ann Anderson-Berry

**Affiliations:** 1 Department of Pediatrics, University of Nebraska Medical Center, Omaha, NE 68198, USA; 2 Medical Nutrition Education Program, College of Allied Health, University of Nebraska Medical Center, Omaha, NE 68198, USA

**Keywords:** poverty, education, insurance, omega-3, eicosapentaenoic acid, pregnancy

## Abstract

Omega-3 (*n*-3) polyunsaturated fatty acids (PUFAs) play a crucial role in fetal growth and neurodevelopment, while omega-6 (*n*-6) PUFAs have been associated with adverse pregnancy outcomes. Previous studies have demonstrated that socioeconomic status (SES) influences dietary intake of *n*-3 and *n*-6 PUFAs, but few studies have evaluated the association between maternal and cord plasma biomarkers of PUFAs and socioeconomic markers. An IRB-approved study enrolled mother–infant pairs (*n* = 55) at the time of delivery. Maternal and cord plasma PUFA concentrations were analyzed using gas chromatography. Markers of SES were obtained from validated surveys and maternal medical records. Mann–Whitney U tests and linear regression models were utilized for statistical analysis. Maternal eicosapentaenoic acid (EPA) (*p* = 0.02), cord EPA (*p* = 0.04), and total cord *n*-3 PUFA concentrations (*p* = 0.04) were significantly higher in college-educated mothers vs. mothers with less than a college education after adjustment for relevant confounders. Insurance type and household income were not significantly associated with *n*-3 or *n*-6 PUFA plasma concentrations after adjustment. Our findings suggest that mothers with lower educational status may be at risk of lower plasma concentrations of *n*-3 PUFAs at delivery, which could confer increased susceptibility to adverse pregnancy and birth outcomes.

## 1. Introduction

Omega-3 (*n*-3) polyunsaturated fatty acids (PUFAs), including α-linolenic acid (ALA), docosahexaenoic acid (DHA), and eicosapentaenoic acid (EPA), have anti-inflammatory properties and provide many health benefits throughout the lifespan. During pregnancy, *n*-3 PUFAs play a crucial role in fetal growth, immunity, and neurodevelopment [1,2,3]. Studies have shown that increased maternal dietary intake of *n*-3 PUFAs during pregnancy has been associated with decreased risk of both preterm birth and infant low birthweight [4,5]. Furthermore, *n*-3 PUFA intake has been reported to ameliorate pregnancy-related co-morbidities such as pre-eclampsia and post-partum depression [6,7]. Conversely, omega-6 (*n*-6) PUFAs, including linoleic acid (LA) and arachidonic acid (AA), have pro-inflammatory properties. Increased serum concentrations of *n*-6 PUFAs have been observed in pregnancy-related conditions including pre-eclampsia, gestational diabetes mellitus, fetal growth restriction, and preterm delivery [8,9]. 

Humans largely depend on dietary intake of ALA, EPA, and DHA to obtain adequate amounts of these nutrients [10]. Food sources rich in *n*-3 PUFAs include fish, nuts, and leafy green vegetables, whereas *n*-6 PUFAs can be found in vegetable oils; hydrogenated oils, nuts; seeds; and animal products such as meat, dairy, and eggs [11,12,13,14]. ALA is an essential *n*-3 PUFA that serves as a precursor to EPA and DHA. The conversion from ALA to EPA and DHA occurs at a low rate in humans (<8% for ALA to EPA and <4% for ALA to DHA), making it difficult to obtain adequate amounts of EPA and DHA through dietary intake of ALA [15,16,17,18,19]. Maternal dietary intake of *n*-3 PUFAs is especially crucial during pregnancy, as the fetus solely relies on maternal nutrient supply in utero [1,20]. Despite the importance of these compounds during pregnancy, we recently reported lower intake of *n*-3 PUFAs in both pregnant women and in non-pregnant females, suggesting that these populations are at elevated risk of *n*-3 PUFA insufficiency [18,21]. For example, we previously reported that the average intake of EPA + DHA in a group of women of childbearing age (*n* = 7266) was 89.0 mg/day, which is approximately 30% of the recommended daily level [22]. Similar findings were reported in a study by Richter et al., demonstrating inadequate *n*-3 PUFA intake of only 170 mg/day (out of 300–500 mg/day recommended) based on an NHANES analysis studying intake in pregnant and or lactating women at least 13 years of age or older in the United States [5,23]. 

The relative intake of *n*-6 PUFAs compared to *n*-3 PUFAs is also important to evaluate when assessing fatty acid status. *n*-3 and *n*-6 PUFAs compete for enzymatic degradation by the shared enzymes fatty acid desaturases (FADSs) 1 and 2 [24]. Thus, increased substrate of either *n*-3 or *n*-6 PUFAs may impact the metabolism of the other. The relationship between *n*-6 and *n*-3 fatty acids in the diet has been reported as a ratio in the literature. The *n*-6:*n*-3 PUFA intake ratio in humans during evolution was described as 1–2:1 and has continued to increase in western diets over the years, ranging anywhere from 15 to 50:1 at present [13,25]. This elevated *n*-6:*n*-3 ratio has been associated with an increased risk of several disease states during pregnancy and early life, including poor fetal neurodevelopment, attention deficit hyperactivity disorder (ADHD) in offspring, post-partum depression, and maternal obesity [3,6,13]. 

Health disparities are also a concern with regard to dietary PUFA intake, as the literature describes that socioeconomic factors may impact *n*-3 and *n*-6 PUFA intake. Specifically, decreased *n*-3 PUFA intake has been demonstrated in socioeconomically disadvantaged groups, including those with lower educational attainment and lower annual income [2,22]. In contrast, increased *n*-6 PUFA intake has been observed in mothers with lower annual incomes [26]. Living in food deserts, food insecurity, knowledge gaps, and access barriers in these populations may also be contributing to potential inequities. Inadequate *n*-3 PUFA intake and increased *n*-6 PUFA consumption in these vulnerable groups may lead to negative health outcomes, which may be compounded in individuals who are already at risk, such as pregnant women. 

Circulating concentrations of *n*-3 and *n*-6 PUFAs are a biomarker for intake and may better reflect actual status than studies of intake alone [21,26]. Despite this, most studies evaluating the impact of social drivers of health on fatty acid status have relied on measurements of dietary intake. Thus, the objective of this research was to determine if maternal socioeconomic status (SES) impacted a validated biological measure of fatty acid status, which is a current gap in the literature. 

## 2. Materials and Methods

### 2.1. Participant Recruitment and Biological Sample Collection 

Ethical approval for this study was obtained from the International Review Board (IRB) of University of Nebraska Medical Center (IRB #112-15-EP). Mother–infant dyads were recruited at Nebraska Medicine, an academic medical center in Omaha, Nebraska, USA. Written informed consent was acquired from the mother prior to participation. Inclusion criteria included women ≥19 years of age who were admitted to the Labor and Delivery service at Nebraska Medicine during the study period and who delivered at least one live-born infant. Exclusion criteria included infants deemed wards of the state; fetal congenital abnormalities; inborn errors of metabolism; and other disorders impacting fatty acid metabolism, such as renal, hepatic, or metabolic diseases. 

Maternal and umbilical cord blood samples were collected in K2 EDTA tubes during routine labs upon admission for delivery. Whole blood samples were protected from heat and light to prevent degradation of unstable molecules. Samples were promptly processed into aliquots of plasma by study personnel and subsequently stored at −80 °C. 

### 2.2. Polyunsaturated Fatty Acid Laboratory Analysis 

Plasma samples (*n* = 55 maternal and *n* = 55 cord) were analyzed for *n*-3 PUFAs including α-linolenic acid (ALA), docosahexaenoic acid (DHA), *n*-3 docosapentaenoic acid (DPA), and eicosapentaenoic acid (EPA), and total *n*-3 PUFAs (sum of ALA, EPA, DPA, and DHA), as well as the *n*-6 PUFAs arachidonic acid (AA) and linoleic acid (LA), and total *n*-6 PUFAs (sum of AA, LA, *n*-6 docosapentaenoic acid, eicosadienoic acid, dihomo-γ-linolenic acid, and docosatetraenoic acid). Quantitation of these nutrients was conducted using gas chromatography with flame ionization detection (GC-FID) at OmegaQuant Analytics LLC (Sioux Falls, SD, USA). Plasma was transferred to a screw-cap glass vial, and boron trifluoride–methanol (BTM) solution (methanol containing 14% boron trifluoride, toluene, methanol; 35:30:35 *v/v/v*; Sigma-Aldrich, St. Louis, MO, USA) and an internal standard were added. The vial was briefly vortexed and heated in a bath at 100 °C for 45 min. After cooling, hexane (Merck KGaA, Darmstadt, Germany) and high-performance liquid chromatography-grade water were added. The tubes were recapped, vortexed, and centrifuged to separate layers. An aliquot of the hexane layer was transferred to a GC vial. GC was conducted using a GC-2010 Gas Chromatograph (Shimadzu Corporation, Columbia, MD, USA) equipped with an SP-2560 100 m fused silica capillary column (0.25 mm in internal diameter, 0.2 um in film thickness; Supelco, Bellefonte, PA, USA). Fatty acids were identified by comparison with a standard mixture of fatty acids (GLC OQ-A; NuChek Prep, Elysian, MN, USA), which was also used to determine individual fatty acid calibration curves. 

### 2.3. Demographic Data and Questionnaires 

Demographic data included maternal age, maternal race, and infant sex. Infant clinical data included delivery mode (vaginal vs. cesarean), birth weight (grams, g), corrected gestational age at birth (weeks), birth length (centimeters, cm), birth head circumference (cm), and neonatal intensive care unit (NICU) admission. Maternal clinical data included pre-eclampsia, smoking status, and pre-pregnancy body mass index (BMI). Maternal BMI was obtained from the mother’s electronic medical records (EMRs) when available and was otherwise obtained via survey responses. The remaining clinical data were collected from the EMRs. 

Questionnaires were administered to the mother by trained study personnel during admission. Maternal dietary intake was assessed with the Harvard Food Frequency Questionnaire (FFQ), a quantitative survey that calculates daily nutrient intake based on reported diet over the last year, which is validated in pregnant patients [22]. An SES questionnaire collected information on annual household income, household size prior to delivery of the infant, and maternal educational attainment. Maternal insurance payor was obtained from the electronic medical records. Markers of maternal SES utilized in statistical analysis included private vs. public insurance (with private insurance serving as a surrogate for higher SES), annual income ≤150% of the poverty line vs. >150% of the poverty line, and college degree earners vs. those with less than a college education [23]. 

### 2.4. Statistical Analysis 

Descriptive statistics were generated for all variables and included medians and interquartile ranges (IQRs) for continuous variables, and frequencies (*n*) and percentages (%) for categorical variables. Total *n*-3 PUFA intake, total *n*-6 PUFA intake, and *n*-6:*n*-3 PUFA intake ratios were analyzed with and without supplementation. The *n*-6:*n*-3 PUFA intake ratio was quantified by dividing total *n*-6 PUFA intake by total *n*-3 PUFA intake. Similarly, the *n*-6:*n*-3 PUFA ratios for plasma markers were calculated by dividing total plasma *n*-6 PUFA concentrations by total plasma *n*-3 PUFA concentrations. The Mann–Whitney U test was used to assess differences in maternal and cord plasma PUFA concentrations between binary categories of SES. The Mann–Whitney U test was also utilized to compare maternal dietary intake of PUFAs among SES groups. A one-sample Wilcoxon signed-rank test was used to compare maternal dietary intake of *n*-3 and *n*-6 PUFAs to the recommended intake. Medians and IQRs were utilized to describe our results, as our data did not follow a parametric distribution. Linear regression models were used to adjust significant associations in the univariate analysis for maternal age and smoking status (never vs. former/current smoker). Nutrient plasma values were natural-log-transformed to meet the assumptions of the regression models. IBM SPSS Statistics 29 software (IBM Corp., New York, NY, USA) was utilized for statistical analyses. A *p*-value < 0.05 was considered statistically significant.

## 3. Results

### 3.1. Baseline Characteristics

A total of 55 mother–infant pairs were enrolled in this study. Median maternal age was 30 years (24–34); median birth corrected gestational age was 39.3 weeks (36.7–40). In terms of race/ethnic group, we found that 58.2% of mothers were White, 20% were Black or African American, 10.9% were Hispanic, 1.8% were Asian or Pacific Islander, and 9.1% identified as other or unknown. In this cohort, 54.5% of mothers had private insurance, while the remaining 45.5% of mothers had public insurance. The baseline characteristics of the maternal and infant cohort including other socioeconomic markers are summarized in Table 1. Median nutrient concentrations in maternal and cord blood at the time of delivery are shown in Table 2 and Table 3, respectively. Consistently with previous findings, cord plasma PUFA concentrations were lower than maternal plasma PUFA concentrations in our cohort [27].

### 3.2. Maternal Education Level, and Plasma n-3 and n-6 PUFA Concentrations

**Figure 1:**
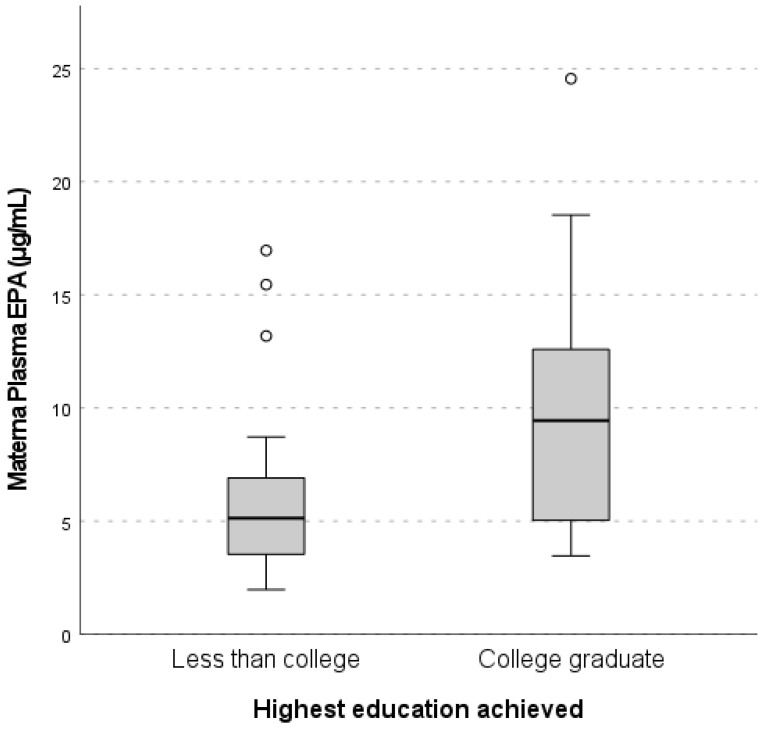
Median maternal plasma eicosapentaenoic acid (EPA) concentrations by education level. Quantitation of EPA in maternal plasma was conducted with gas chromatography. Maternal educational attainment was obtained with a questionnaire administered to the mother by trained study personnel. Differences in maternal plasma EPA concentrations between binary categories of socioeconomic status (SES) were assessed with the Mann–Whitney U test. Maternal plasma EPA concentrations were significantly higher in college graduates vs. those with less than a college education (9.44 µg/mL vs. 5.13 µg/mL, *p* = 0.01). Gray boxes represent the IQRs and include median plasma levels, while white dots include plasma levels that fell more than 1.5 times the IQR above the 75th percentile.

Out of 55 participants, 48 subjects completed the FFQ and the SES questionnaire. Twelve mothers reported their annual income as “Unknown”, and these were excluded from the income analyses. Our comparison of maternal nutrient concentrations between college-educated mothers versus mothers with less than a college education showed a significant difference in maternal EPA concentrations (9.44 µg/mL vs. 5.13 µg/mL, *p* = 0.01) (Figure 1). After adjusting for maternal age and smoking status, having a college degree predicted a 0.47 µg/mL increase in natural-log-transformed maternal EPA (equivalent to a 1.60 µg/mL increase in non-transformed maternal EPA) compared with women with less than a college degree (β = 0.47, 95% CI 0.09–0.86, *p* = 0.02). In cord plasma, significantly higher *n*-3 PUFA concentrations were present in college-educated mothers compared with less-than-college-educated mothers for cord EPA (1.88 µg/mL vs. 1.40 µg/mL, *p* = 0.01), cord DHA (37.96 µg/mL vs. 32.80 µg/mL, *p* = 0.01), and total cord *n*-3 PUFAs (44.23 µg/mL vs. 39.34 µg/mL, *p* = 0.02) (Table 3). After adjustment for maternal age and smoking status, maternal educational level continued to be significantly associated with natural-log-transformed cord EPA (β = 0.17, 95% CI 0.01–0.33, *p* = 0.04) and cord total *n*-3 PUFA concentrations (β = 0.25, 95% CI 0.02–0.49, *p* = 0.04) but not cord DHA concentrations (β = 0.25, 95% CI = −0.12–0.61, *p* = 0.18). There were no significant differences in maternal or cord plasma concentrations of *n*-6 PUFAs when comparing groups by education level. 

### 3.3. Maternal Economic Level, and Plasma n-3 and n-6 PUFA Concentrations

**Figure 2:**
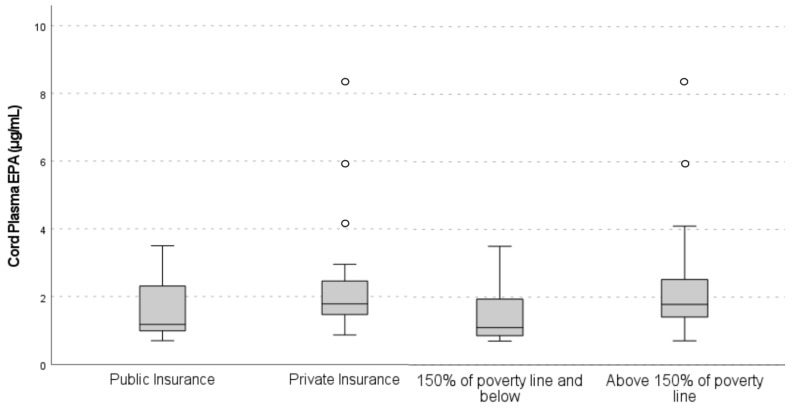
Median cord plasma EPA concentrations by insurance type and annual income. Quantitation of EPA in cord plasma was conducted with gas chromatography. Maternal insurance type was obtained from the electronic medical records. The Mann–Whitney U test was used to assess differences in cord plasma EPA concentrations between binary categories of SES. Cord plasma EPA concentrations were significantly higher in mothers with private vs. public insurance (1.79 µg/mL vs. 1.18 µg/mL, *p* = 0.02). This relationship was attenuated after adjustment for maternal age and smoking status (β = 0.13, 95% CI −0.03–0.28, *p* = 0.10). Cord plasma EPA concentrations were significantly higher in mothers with higher annual income vs. lower annual income (1.79 µg/mL vs. 1.10 µg/mL, *p* = 0.03). After adjustment for maternal age and smoking status, there was no longer a significant relationship between annual income and cord EPA (β = 0.14, 95% CI −0.05–0.34, *p* = 0.13). Gray boxes represent the IQRs and include median plasma levels, while white dots include plasma levels that fell more than 1.5 times the IQR above the 75th percentile.

When comparing groups by insurance type, median cord EPA concentrations were significantly higher in those with private insurance compared with mothers with public insurance (1.79 µg/mL vs. 1.18 µg/mL, *p* = 0.02) (Figure 2). After adjustment for maternal age and smoking status, there was no longer a significant relationship between insurance type and cord EPA (β = 0.13, 95% CI −0.03–0.28, *p* = 0.10). Maternal plasma nutrient concentrations were not significantly different between mothers with private insurance compared with public insurance for *n*-3 and *n*-6 PUFAs. 

There was no significant difference in maternal plasma nutrient concentrations when comparing income groups. However, significantly higher cord plasma EPA concentrations were present in mothers whose income was >150% of the poverty line (1.79 µg/mL vs. 1.10 µg/mL, *p* = 0.03) compared with those with annual incomes ≤150% of the poverty line (Figure 2). After adjustment for maternal age and smoking status, this relationship was attenuated (β = 0.14, 95% CI −0.05–0.34, *p* = 0.13). 

### 3.4. Maternal Dietary Intake of n-3 and n-6 PUFAs, and Intake Comparison among SES Categories

Median maternal intake of ALA was 1.29 g/day (0.94–1.74) in this cohort, which was below the adequate intake recommended by the National Academy of Medicine of 1.4 g/day during pregnancy [11], although this was not statistically significant (*p* = 0.59). Median maternal EPA intake was 0.02 g/day (0.00–0.06), and median maternal DHA intake was 0.07 g/day (0.03–0.17). Median EPA + DHA intake was significantly below the recommended intake for the cohort based on guidelines put forth by the World Health Organization of 300 mg/day in pregnancy (*p* = 0.01) [5]. 

Maternal intake data are included in Table 4. There were no significant differences in maternal dietary intake of *n*-3 or *n*-6 PUFAs according to education level or insurance type, regardless of supplementation status. Although there were no significant differences in *n*-3 or *n*-6 PUFA intake between income groups, total *n*-3 PUFA intake (no supplementation) approached a significant difference between those with higher annual income and the lower-income group (1.30 g/day vs. 1.75 g/day, *p* = 0.07).

## 4. Discussion

In the present study, we evaluated the impact of social drivers of health, such as maternal education, annual income, and insurance type, on fatty acid status, utilizing plasma concentrations as a biomarker for PUFA status in mother–infant dyads. Several previous analyses have investigated the association between SES, and dietary intake of *n*-3 and *n*-6 PUFAs, but few studies have assessed the relationship between SES and plasma concentrations of these nutrients as we did in this study. Our data demonstrate that higher maternal educational attainment is associated with higher concentrations of *n*-3 PUFAs in maternal and cord plasma. Additional literature is in agreement with the positive relationship between medium-to-high educational attainment and higher maternal plasma concentrations of *n*-3 PUFAs throughout pregnancy. For instance, Aparicio et al. demonstrated significantly higher maternal serum concentrations of EPA, DHA, and total *n*-3 PUFAs during the third trimester of pregnancy among women with high versus low educational attainment [28]. Stark et al. also recognized significantly greater serum concentrations of DHA in pregnant women with higher educational attainment (β 1.03, *p* = 0.041) [29]. Moreover, our study also demonstrated that higher maternal education was significantly associated with higher cord plasma concentrations of EPA and total *n*-3 PUFAs; these findings in cord plasma are novel. Interestingly, maternal dietary intake of EPA did not significantly differ among mothers when compared by education level. 

While significant differences in maternal plasma concentrations of *n*-3 PUFAs were observed between groups when comparing education level, no significant differences in maternal plasma PUFA concentrations were present between groups according to income level or insurance type. Contrary to our results, a study by Pinto et al. described a significant negative association between monthly income and the *n*-6:*n*-3 PUFA ratio in maternal serum (β −0.002, *p* = 0.013) [26]. The association between income level and serum concentrations of *n*-3 PUFAs has also been analyzed in additional populations. For example, Cohen et al. assessed the impact of SES on serum concentrations of *n*-3 PUFAs in patients with coronary artery disease. This study demonstrated that serum EPA + DHA concentrations were 14% lower in participants with an annual household income <USD 20,000 compared with those whose annual income was >USD 50,000 (4.06 g/100 g total fatty acids vs. 4.70 g/100 g total fatty acids, *p* < 0.001) [30]. While the impact of insurance type on dietary intake and plasma concentrations of *n*-3 PUFAs has not been well described, a study by Lim et al. demonstrated that having private health insurance was significantly associated with increased intake of fruits (OR 1.73; 95% CI 1.39–2.17) and vegetables (OR 1.50; 95% CI 1.18–1.90) in an Australian cohort [31]. Their study suggests that insurance type may influence dietary patterns and subsequently plasma nutrient concentrations in that region. Several other factors, including genetic components, maternal adaptive mechanisms, nutrient bioavailability, and access barriers, may also impact the differences observed in plasma concentrations among SES groups [8,9,10,11,18,28,32,33].

Genetic factors impact the metabolism of *n*-3 PUFAs, which may result in differential plasma concentrations and health outcomes, regardless of dietary intake [34]. Fatty acid desaturase (FADS) 1 and FADS2 are rate-limiting enzymes in the conversion of ALA to EPA and DHA. Single-nucleotide polymorphisms of FADS1 and FADS2 exist in humans, and research has shown that variants in these enzymes influence plasma concentrations of *n*-3 PUFAs in pregnancy [24,32]. Furthermore, the literature supports that unique socioeconomic factors may result in differential gene expression in individuals, which may ultimately impact the utilization of *n*-3 PUFAs [35,36]. This concept suggests that individuals may require different dietary sources and amounts of *n*-3 PUFAs to achieve similar biological effects [37]. A mother’s metabolic state also influences placental mechanisms that protect offspring from low concentrations of *n*-3 PUFAs when maternal dietary intake is low [38]. The efficiency of these placental functions may differ among individuals, ultimately resulting in different cord plasma nutrient concentrations. These data suggest that *n*-3 PUFA concentrations can also be related with genetic factors such as single-nucleotide polymorphisms in our maternal–fetal cohort and requires further investigations. 

Another possible explanation for the observed association between higher SES and elevated plasma *n*-3 PUFAs is that access to food sources of *n*-3 PUFAs may have differed among SES groups, resulting in unique nutrient bioavailability [19]. Mothers with lower SES may have limited means to access foods that are rich in *n*-3 PUFAs, such as fresh fish and nuts, and may opt for lower-cost alternatives, which may contain *n*-3 PUFAs in a form that is less readily available for metabolism [26]. Not only does the primary food source matter in terms of nutrient bioavailability, but the concomitant intake of other molecules, such as calcium, can impact the breakdown of *n*-3 PUFAs [33]. The literature supports that women with higher educational attainment may be more knowledgeable about nutrient-dense foods and the utility of a healthy diet in the modulation of disease states [39,40]. Having access to this information may have impacted maternal food choices in the higher-SES group, potentially resulting in intake of foods with more accessible *n*-3 PUFAs. This may explain why although there was no difference in intake of *n*-3 and *n*-6 PUFAs among SES groups in our cohort, plasma concentrations of *n*-3 PUFAs were significantly different. 

Contrary to our hypothesis, maternal dietary intake of PUFAs did not significantly differ among SES groups in our cohort. Multiple studies analyzing NHANES data from 2003–2014 have demonstrated that socioeconomic factors, specifically higher maternal education level and higher annual income, were associated with elevated maternal dietary intake of *n*-3 PUFAs [2,22]. Similarly, Jahns et al. described a significant association between higher educational attainment and increased seafood consumption, providing a higher amount of dietary *n*-3 PUFAs for the higher-SES group [41]. These studies were conducted in a broader cohort than that in our study, with two of them representing the entire U.S. civilian, non-institutionalized population, while the other specifically focused on women of childbearing age. Other research conducted in populations more similar to ours, specifically in cohorts of pregnant women, discovered no link between educational attainment and *n*-3 PUFA intake, in agreement with our findings [28,42]. One explanation of our findings may be the smaller sample size of this cohort. 

Most notably, the lack of difference seen in dietary intake of *n*-3 PUFAs among SES groups may be attributable to the low concentrations of *n*-3 PUFA intake for the entire cohort, regardless of SES. The median maternal intake of ALA in our cohort was 1270 mg/day, which is below recommended as per adequate intake of 1400 mg/day in pregnancy [43]. While there is no universal recommendation for dietary intake of EPA + DHA, several organizations have developed their own guidelines. The Joint Food and Agriculture Organization of the United Nations/World Health Organization Expert Consultation on Fats and Fatty Acids in Human Nutrition 2010 provided maternal intake recommendations of at least 300 mg/day of EPA + DHA in pregnancy, with at least 200 mg/day in the form of DHA for optimal fetal development [43,44]. In the same year, the European Food Safety Authority (EFSA) published guidelines opting for 250 mg/day of EPA + DHA in adults, with an additional recommended 100–200 mg/day of DHA in pregnancy and lactation [5]. The American College of Obstetricians and Gynecologists’ (ACOG’s) most recent guidelines, updated in 2020, suggest that mothers intake 2–3 servings of fish weekly, providing approximately 250–375 mg of EPA + DHA per day in pregnancy [5,45]. As per these expert recommendations, our study observed that the median EPA + DHA intake in our maternal cohort was 90 mg/day, which was significantly lower than the recommendation of 300 mg/day (*p* = 0.01). Therefore, future studies in larger cohorts are indicated to more completely distinguish how socioeconomic status impacts biological concentrations of these fatty acids and perinatal health outcomes. 

We additionally analyzed the *n*-6:*n*-3 PUFA ratio in our study, a marker that helps to describe the relative relationship between *n*-6 and *n*-3 PUFAs in the maternal diet and plasma samples. Although the median *n*-6:*n*-3 PUFA intake ratio did not significantly differ among SES groups in our cohort, both groups had ratios above the optimal range of 1–2:1 [3,25]. The median *n*-6:*n*-3 PUFA intake ratio (no supplementation) in our cohort was 9.1:1, which is over four times higher than the recommendation. It is possible that increased concentrations of *n*-6 PUFAs—combined with overall inadequate dietary intake of *n*-3 PUFAs—hindered the metabolism of *n*-3 PUFAs in our entire cohort, with the lower-SES group being disproportionately affected. The median *n*-6:*n*-3 PUFA ratio was also elevated in both maternal and cord plasma but did not significantly differ among SES groups. An elevated *n*-6:*n*-3 PUFA ratio conferred an increased risk of maternal and infant comorbidities, including neurodevelopmental delay, post-partum depression, and elevated maternal body mass index, in our entire cohort [3,6,13]. Notably, 17 out of 55 infants were admitted to the NICU despite having a median corrected gestational age of 39.3 weeks in our cohort. While we are not able to suggest a causal relationship between an elevated *n*-6:*n*-3 PUFA ratio and NICU admission in our study, it is worth recognizing that a high proportion (30.9%) of our study population required a higher level of care. 

There are numerous limitations to this study, which include the study being conducted at a single academic medical institution in the Midwest United States, with a majority of study participants identifying as non-Hispanic Whites. The demographics of our study population may impact the generalizability of our results. Additionally, our small sample size creates less of an impact; thus, future investigations should attempt to replicate these results in a larger, more heterogenous sample. Another area for improvement involves the specimens that we had available for analysis. Specifically, we did not assess the red blood cell (RBC) fatty acid profile or RBC omega-3 index. These markers would have been helpful in corroborating the FFQ data, as both indices reflect long-term dietary exposures to fatty acids [46]. Additionally, we discuss that genetic factors impact PUFA metabolism but were unable to identify genetic variants in maternal DNA that may be involved in the PUFA metabolic pathway. Genetic testing on maternal and cord plasma samples was beyond the scope of this study but should be addressed in future iterations. Another limitation of this study lies in the use of participant surveys, which are a feasible method for collecting data but remain vulnerable to response bias and non-response bias [47]. Although the FFQ has been validated in pregnant women, this survey may be particularly vulnerable to biased responses, as this survey is not optimized to capture variable nutrient bioavailability across similar foods, participant knowledge of food nutritional content, or other factors that affect food choices. Future studies should continue to optimize available dietary assessment tools to more accurately capture maternal diets. Additionally, we were unable to obtain survey results for all participants due to multiple reasons. Some mothers declined participation in specific questionnaires; some were discharged prior to survey completion; and some were lost to follow-up. Additional modes of distribution, such as electronic surveys, could be useful in improving questionnaire participation in future studies.

## 5. Conclusions

In this study, we assessed the impact of socioeconomic status on dietary intake of *n*-3 and *n*-6 PUFAs, as well as the association between SES, and maternal and cord plasma concentrations of *n*-3 and *n*-6 PUFAs in pregnancy. Overall, *n*-3 PUFA intake was low in our cohort. There were no significant differences in *n*-3 or *n*-6 PUFA intake among SES categories. SES did have an impact on plasma concentrations of *n*-3 PUFAs. Specifically, higher maternal educational attainment was associated with significantly higher concentrations of EPA in maternal and cord plasma as well as total *n*-3 PUFAs in cord plasma after adjusting for maternal age and smoking status. Our findings suggest that mothers with lower educational attainment may be at risk of lower plasma concentrations of *n*-3 PUFAs during pregnancy, which may confer an increased risk of adverse health outcomes in socioeconomically disadvantaged populations. Plasma *n*-3 PUFA concentrations in pregnancy may be an under-recognized health disparity in the United States, although further research in a larger, more heterogenous sample is warranted to determine the scope of this public health issue. The results of our study may be beneficial in identifying groups that are at risk of low plasma concentrations of *n*-3 PUFAs in pregnancy, ultimately providing the opportunity to educate patients on the importance of adequate nutrition in the peripartum period. Furthermore, our analysis and future iterations may help to guide dietary recommendations of *n*-3 PUFAs during pregnancy in order to optimize maternal and infant outcomes. 

## Figures and Tables

**Table 1:** Maternal and infant demographics and clinical characteristics.

Participant Characteristic	*N*	Median (IQR)
Maternal age (years)	55	30 (24–34)
Corrected gestational age at birth (weeks)	55	39.3 (36.7–40)
Infant birth weight (g)	55	3313 (2540–2714)
Infant birth head circumference (cm)	55	33.7 (32.4–35.6)
Infant birth length (cm)	54	49.0 (46.7–50.8)
Maternal pre-pregnancy BMI (kg/m^2^)	51	27.5 (22.0–33.0)
	***N***	**%**
Infant sex		
Male	28	50.9
Female	27	49.1
Maternal race		
White	32	58.2
African American	11	20.0
Hispanic	6	10.9
Asian or Pacific Islander	1	1.8
Other/unknown	5	9.1
Pre-eclampsia		
Yes	8	14.5
No	47	85.5
Maternal diabetes		
Yes	5	9.1
No	50	90.9
Infant NICU admission		
Yes	17	30.9
No	38	69.1
Maternal smoking status		
Never smoked	44	80.0
Current smoker	1	1.8
Former smoker	10	18.2
Maternal Insurance Type		
Public	25	45.5
Private	30	54.5
Maternal highest education		
Less than college	26	47.3
College graduate	22	40.0
Did not complete survey	7	12.7
Maternal annual income		
≤150% of poverty line	10	18.2
>150% of poverty line	26	47.3
“Unknown”	12	21.8
Did not complete survey	7	12.7

**Table 2:** Maternal plasma nutrient concentrations (µg/mL) at delivery by socioeconomic status.

Nutrient	*N*	Median Plasma Level (µg/mL; IQR)—Entire Cohort	*N*	Median Plasma Level (µg/mL; IQR)—Higher-Education Group	*N*	Median Plasma Level (µg/mL; IQR)—Lower-Education Group		
α-Linolenic acid (ALA)	55	30.7 (25.6–39.5)	22	29.59 (25.21–39.41)	26	33.87 (26.83–41.29)		
Arachidonic acid (AA)	55	242.4 (25.6–39.5)	22	253.64 (217.79–288.36)	26	219.28 (195.64–295.71)		
Docosahexaenoic acid (DHA)	55	62.5 (51.3–80.6)	22	66.84 (58.11–86.95)	26	59.56 (46.97–75.59)		
Docosapentaenoic-n3 acid (DPA)	55	9.3 (7.4–12.2)	22	9.90 (8.33–12.77)	26	9.36 (6.96–11.97)		
Eicosapentaenoic acid (EPA)	55	5.57 (3.9–9.5)	22	9.44 (5.02–12.60)	26	5.13 (3.45–6.98)		
Linoleic acid (LA)	55	1349.6 (1191.2–1594.9)	22	1331.41 (1208.76–1617.27)	26	1341.01 (1180.72–1586.98)		
*n*-6:*n*-3 PUFA ratio	55	15.32 (13.4–17.6)	22	14.80 (12.43–16.55)	26	15.83 (13.42–19.10)		
Total *n*-3 PUFAs	55	114.6 (90.1–139.5)	22	125.96 (99.03–148.03)	26	109.81 (86.47–125.83)		
Total *n*-6 PUFAs	55	1733.1 (1480.9–1989.2)	22	1753.66 (1503.00–2030.11)	26	1709.08 (1476.47–1911.24)		
**Nutrient**	***N***	**Median Plasma Level (µg/mL; IQR)—Higher-Income Group**	***N***	**Median Plasma Level (µg/mL; IQR)—Lower-Income Group**	***N***	**Median Plasma Level (µg/mL; IQR)—Private** **Insurance**	***N***	**Median Plasma Level (µg/mL; IQR)—Public** **Insurance**
ALA	26	29.59 (25.21–39.41)	10	36.75 (30.04–40.96)	30	28.70 (23.53–40.32)	25	34.65 (28.30–40.08)
AA	26	244.19 (199.90–302.93)	10	261.13 (192.78–387.89)	30	237.17 (193.92–288.36)	25	259.87 (204.23–302.36)
DHA	26	62.44 (55.93–83.28)	10	65.07 (41.17–95.02)	30	62.16 (50.87–83.28)	25	68.56 (53.01–80.41)
DPA	26	10.12 (8.27–12.64)	10	8.03 (7.28–12.57)	30	9.15 (7.20–12.10)	25	9.86 (7.27–12.58)
EPA	26	7.53 (4.88–11.77)	10	4.22 (2.92–8.48)	30	5.68 (4.33–11.44)	25	5.55 (3.62–7.81)
LA	26	1331.41 (1208.76–1596.27)	10	1472.69 (1290.51–1807.67)	30	1331.41 (1119.97–1617.27)	25	1443.15 (1217.03–1589.63)
*n*-6:*n*-3 PUFA ratio	26	15.08 (12.43–17.00)	10	17.85 (11.75–20.08)	30	15.38 (13.28–17.32)	25	15.28 (13.28–18.15)
Total *n*-3 PUFAs	26	118.89 (93.68–146.20)	10	108.94 (86.23–152.86)	30	110.84 (88.47–141.15)	25	118.09 (91.05–142.39)
Total *n*-6 PUFAs	26	1753.66 (1503.00–1993.82)	10	1795.99 (1652.90–2268.38)	30	1678.78 (1446.64–2030.11)	25	1769.63 (1607.94–1953.95)

**Table 3:** Cord plasma nutrient concentrations (µg/mL) at delivery by socioeconomic status.

Nutrient	*N*	Median Plasma Level (µg/mL; IQR)— Entire Cohort	*N*	Median Plasma Level (µg/mL; IQR)—Higher Education-Group	*N*	Median Plasma Level (µg/mL; IQR)—Lower-Education Group		
ALA	55	1.9 (1.2–3.1)	22	2.05 (1.28–2.96)	26	1.82 (0.95–3.07)		
AA	55	154.6 (127.7–178.3)	22	157.79 (130.59–179.47)	26	141.84 (124.19–176.68)		
DHA	55	34.9 (28.3–42.6)	22	37.96 (34.31–49.69)	26	32.80 (25.59–37.68)		
DPA	55	2.5 (1.8–3.5)	22	2.69 (2.15–4.64)	26	2.37 (1.58–3.07)		
EPA	55	1.6 (1.1–2.4)	22	1.88 (1.41–2.63)	26	1.40 (1.00–1.81)		
LA	55	135.4 (135.4–166.3)	22	135.61 (117.26–190.70)	26	122.42 (98.72–161.52)		
*n*-6:*n*-3 ratio	55	8.6 (6.8–9.6)	22	7.92 (6.52–9.07)	26	8.77 (6.96–9.65)		
Total *n*-3 PUFAs	55	40.4 (32.9–53.5)	22	44.23 (39.29–59.19)	26	39.34 (29.67–44.74)		
Total *n*-6 PUFAs	55	347.9 (283.2–400.7)	22	349.63 (308.21–402.99)	26	321.34 (269.51–388.93)		
**Nutrient**	***N***	**Median Plasma Level (µg/mL; IQR)—** **Private Insurance**	***N***	**Median Plasma Level (µg/mL; IQR)—Public Insurance**	***N***	**Median Plasma Level (µg/mL; IQR)—** **Higher-Income Group**	***N***	**Median Plasma Level (µg/mL; IQR)—** **Lower-Income Group**
ALA	30	1.84 (1.28–3.31)	25	2.06 (1.05–2.93)	26	2.05 (1.26–2.78)	10	1.78 (1.17–3.13)
AA	30	158.82 (133.09–184.10)	25	142.06 (126.61–176.98)	26	157.79 (127.64–178.75)	10	140.16 (117.95–177.71)
DHA	30	35.08 (31.03–49.28)	25	33.47 (25.81–40.03)	26	36.23 (32.40–49.29)	10	36.27 (24.08–43.15)
DPA	30	2.47 (1.91–3.75)	25	2.55 (1.55–3.63)	26	2.56 (2.02–4.64)	10	2.89 (2.31–3.36)
EPA	30	1.79 (1.46–2.48)	25	1.18 (0.99–2.32)	26	1.79 (1.40–2.58)	10	1.10 (0.83–1.96)
LA	30	135.61 (106.61–190.70)	25	124.72 (98.26–157.38)	26	135.48 (109.56–168.67)	10	122.41 (102.97–138.95)
*n*-6:*n*-3 ratio	30	8.55 (6.78–10.55)	25	8.78 (7.10–9.50)	26	8.42 (6.64–9.10)	10	7.87 (5.92–9.65)
Total *n*-3 PUFAs	30	41.28 (36.73–57.15)	25	39.47 (30.28–48.86)	26	43.73 (37.72–59.19)	10	42.19 (29.55–49.14)
Total *n*-6 PUFAs	30	349.02 (308.21–418.42)	25	331.83 (272.21–386.48)	26	349.02 (305.67–401.08)	10	299.40 (275.61–379.95)

**Table 4:** Maternal dietary intake (g/day) of *n*-3 and *n*-6 PUFAs by socioeconomic status.

Nutrient	*N*	Median Intake (g/day; IQR) for Entire Cohort	*N*	Median Intake (g/day; IQR) for Higher-Education Group	*N*	Median Intake (g/day; IQR) for Lower-Education Group	*p*-Value
ALA intake	48	1.29 (0.94–1.74)	22	1.26 (1.12–1.56)	26	1.35 (0.88–1.95)	0.61
AA intake	48	0.14 (0.09–0.18)	22	0.14 (0.10–0.19)	26	0.14 (0.09–0.18)	0.84
DHA intake	48	0.07 (0.03–0.17)	22	0.07 (0.03–0.19)	26	0.07 (0.03–0.17)	0.79
DPA intake	48	0.02 (0.01–0.03)	22	0.02 (0.01–0.03)	26	0.02 (0.01–0.03)	0.77
EPA intake	48	0.02 (0.00–0.06)	22	0.03 (0.01–0.14)	26	0.02 (0.00–0.06)	0.33
LA intake	48	11.99 (8.86–15.60)	22	10.88 (9.07–13.34)	26	12.86 (7.93–19.00)	0.41
*n*-6:*n*-3 PUFA intake ratio (no supplementation)	48	9.12 (8.22–10.08)	22	9.19 (8.21–10.06)	26	9.08 (8.51–10.21)	0.80
*n*-6:*n*-3 PUFA intake ratio (with supplementation)	48	8.95 (7.29–10.06)	22	8.21 (6.67–9.89)	26	9.08 (8.14–10.21)	0.15
Total *n*-3 PUFA intake (no supplementation)	48	1.43 (1.07–1.84)	22	1.30 (1.10–1.54)	26	1.53 (0.94–2.06)	0.33
Total *n*-3 PUFA intake (with supplementation)	48	1.53 (1.09–1.98)	22	1.47 (1.18–1.94)	26	1.61 (0.94–2.06)	0.98
Total *n*-6 PUFA intake (no supplementation)	48	13.09 (9.72–17.11)	22	11.61 (10.09–14.61)	26	14.57 (8.74–21.15)	0.35
Total *n*-6 PUFA intake (with supplementation)	48	13.09 (9.72–17.11)	22	11.61 (10.09–14.61)	26	14.57 (8.74–21.15)	0.36
**Nutrient**	***N***	**Median Intake (g/day; IQR) for Higher-Income Group**	***N***	**Median Intake (g/day; IQR)** **for Lower-Income Group**	***p*-Value**		
ALA intake	26	1.26 (1.05–1.72)	10	1.55 (1.28–1.78)	0.19		
AA intake	26	0.14 (0.09–0.18)	10	0.15 (0.11–0.27)	0.43		
DHA intake	26	0.09 (0.03–0.23)	10	0.07 (0.06–0.25)	0.48		
DPA intake	26	0.02 (0.01–0.03)	10	0.02 (0.01–0.03)	0.90		
EPA intake	26	0.03 (0.01–0.14)	10	0.03 (0.01–0.08)	0.88		
LA intake	26	10.87 (8.99–15.36)	10	13.73 (11.24–15.46)	0.21		
*n*-6:*n*-3 PUFA intake ratio (no supplementation)	26	9.01 (8.21–10.06)	10	8.80 (7.94–9.89)	0.72		
*n*-6:*n*-3 PUFA intake ratio (with supplementation)	26	8.21 (6.76–9.85)	10	8.80 (7.94–9.89)	0.48		
Total *n*-3 PUFA intake (no supplementation)	26	1.30 (1.09–1.75)	10	1.75 (1.45–1.88)	0.07		
Total *n*-3 PUFA intake (with supplementation)	26	1.47 (1.15–2.02)	10	1.75 (1.51–1.88)	0.57		
Total *n*-6 PUFA intake (no supplementation)	26	11.61 (10.02–16.65)	10	15.49 (12.29–17.09)	0.18		
Total *n*-6 PUFA intake (with supplementation)	26	11.61 (10.02–16.65)	10	15.49 (12.29–17.09)	0.19		
**Nutrient**	***N***	**Median Intake (g/day; IQR) for Private** **Insurance**	***N***	**Median Intake (g/day; IQR) for** **Public** **Insurance**	***p*-Value**		
ALA intake	26	1.26 (1.05–1.83)	22	1.35 (0.84–1.73)	0.98		
AA intake	26	0.14 (0.10–0.19)	22	0.14 (0.09–0.18)	0.66		
DHA intake	26	0.07 (0.03–0.23)	22	0.07 (0.03–0.16)	0.73		
DPA intake	26	0.02 (0.01–0.03)	22	(0.02 (0.01–0.03)	0.63		
EPA intake	26	0.02 (0.00–0.11)	22	0.01 (0.00–0.07)	0.59		
LA intake	26	11.01 (9.07–15.36)	22	12.96 (7.58–16.42)	0.85		
*n*-6:*n*-3 PUFA intake ratio (no supplementation)	26	9.05 (10.09–8.20)	22	9.17 (8.49–9.98)	0.90		
*n*-6:*n*-3 PUFA intake ratio (with supplementation)	26	8.21 (6.76–10.07)	22	9.17 (8.49–9.98)	0.17		
Total *n*-3 PUFA intake (no supplementation)	26	1.30 (1.09–1.75)	22	1.53 (0.93–1.87)	0.61		
Total *n*-3 PUFA intake (with supplementation)	26	1.47 (1.15–2.12)	22	1.61 (0.93–1.87)	0.56		
Total *n*-6 PUFA intake (no supplementation)	26	11.91 (10.09–16.65)	22	14.57 (8.20–18.08)	0.76		
Total *n*-6 PUFA intake (with supplementation)	26	11.91 (10.09–16.65)	22	14.57 (8.20–18.08)	0.77		

## Data Availability

Data are available upon request from the corresponding author. These data are not publicly available due to ethical considerations regarding participant confidentiality.
